# Host-dependent effects of *mcr-1* and *mcr-8* on fitness, virulence, and transmission in *Escherichia coli* and *Klebsiella pneumoniae*

**DOI:** 10.3389/fmicb.2026.1908233

**Published:** 2026-08-12

**Authors:** Mengyu Zhou, Yining Ye, Nuoyou Cheng, Huangdu Hu, Xinhong Han, Qing Yu, Yan Jiang, Yunsong Yu, Dongdong Zhao, Jingjing Quan

**Affiliations:** 1 Department of Infectious Diseases, Sir Run Run Shaw Hospital, Zhejiang University School of Medicine, Hangzhou, Zhejiang, China; 2 Zhejiang Provincial Engineering Research Center of Innovative Instruments for Precise Pathogen Detection, Hangzhou, Zhejiang, China; 3 Department of Infectious Diseases, Zhejiang Provincial People's Hospital, Affiliated People's Hospital, Hangzhou Medical College, Hangzhou, Zhejiang, China; 4 Department of Clinical Laboratory, Zhejiang Cancer Hospital, Hangzhou Institute of Medicine (HIM), Chinese Academy of Sciences, Hangzhou, Zhejiang, China

**Keywords:** *Enterobacterales*, fitness cost, host specificity, *mcr-1*, *mcr-8*, virulence

## Abstract

**Introduction:**

As one of the most common groups of clinical pathogens, *Enterobacterales* pose a serious threat to global public health due to their multidrug-resistance. Polymyxin serves as a last-line treatment for infections caused by Carbapenem-resistant *Enterobacterales* (CRE). The emergence and spread of the mobilized colistin resistance (*mcr*) genes, particularly the globally prevalent *mcr-1* and *mcr-8*, have raised significant public health concerns. Epidemiological data reveal a striking host tropism among these genes: *mcr-1* is predominantly found in *Escherichia coli* (83.75%), while *mcr-8* is primarily detected in *Klebsiella pneumoniae* (92.68%). The underlying mechanisms driving this host-specific distribution and its implications for bacterial virulence remain poorly understood.

**Methods:**

This study investigated the host-dependent fitness, transmission, and virulence effects of *mcr-1* and *mcr-8* using a combination of recombinant plasmids transformed into *E. coli* DH5α and *K. pneumoniae* ATCC 13883, alongside clinical isolates.

**Results:**

Our results demonstrate that the biological cost and benefit associated with these plasmids are host-dependent. In *K. pneumoniae*, the *mcr-8* plasmid exhibited enhanced stability and fitness. Conversely, the *mcr-1* plasmid conferred a competitive advantage in *E. coli*. The impact on bacterial virulence in *Galleria mellonella* larvae infection model varied with the host strain.

**Discussion:**

Our findings indicate that the fitness, transmission potential, and virulence modulation conferred by *mcr-1* and *mcr-8* are closely associated with the bacterial host. Specifically, the *mcr-1* plasmid exhibits enhanced transmissibility in *E. coli* compared to *K. pneumoniae*, whereas the *mcr-8* plasmid demonstrates a transmission advantage in *K. pneumoniae* over *E. coli*. The findings of this study clarify that the preferred hosts of *mcr-1* and *mcr-8* plasmids are *E. coli* and *K. pneumoniae*, respectively, and confirm that plasmid fitness, transmissibility, and virulence effects are host-dependent. These results suggest that future development of prevention and control strategies should incorporate differentiated interventions targeting distinct bacterial hosts and plasmid types, in order to more effectively curb the spread of *mcr* genes and extend the clinical utility of polymyxin.

## 1 Introduction

The continuous global increase in multidrug-resistant Gram-negative bacteria, especially the rising detection rate of Carbapenem-resistant *Enterobacterales* (CRE), has become a potential issue for healthcare-associated infections. The World Health Organization has classified CRE as a “critical priority” resistant pathogen, highlighting the global threat of its resistance transmission (Sati et al., 2025). In this severe context, polymyxins, a class of antibiotics once abandoned, have re-entered the clinical arena.

Unfortunately, due to the extensive use and irrational application of polymyxins in both clinical medicine and veterinary medicine, polymyxin-resistant pathogens have rapidly emerged worldwide. In 2015, Chinese researchers first reported the plasmid-mediated polymyxin resistance gene *mcr-1*, a discovery that challenged the conventional understanding that polymyxin resistance was solely mediated by chromosomal mutations. Given its plasmid location, its capacity for horizontal transfer has attracted major concern, further raising anxieties about the future medical environment (Liu et al., 2016). Currently, the *mcr* gene family has expanded to eleven members, designated *mcr-1* through *mcr-11*. Studies on the molecular mechanism have identified that the product encoded by *mcr* genes, MCR, is a phosphoethanolamine transferase. This enzyme catalyzes the modification of lipid A in the lipopolysaccharide of the bacterial outer membrane with phosphoethanolamine, significantly reducing the net negative charge on the bacterial outer membrane surface. This reduction consequently weakens the electrostatic interaction between polymyxin and the outer membrane, leading to the emergence of the resistant phenotype (Aghapour et al., 2019; Liu et al., 2024).

As clinically common pathogenic bacteria, *Enterobacterales* are the primary hosts for *mcr* genes. According to 2024 data from CHINET^1^, *E. coli* and *K. pneumoniae* have ranked as the top two clinically isolated bacterial species, accounting for 18.2 and 13.9% of isolates, respectively.

Global surveillance data from the NCBI National Database of Antibiotic Resistant Organisms (NDARO) reveal a significant host-specific distribution of the *mcr* gene family among different *Enterobacterales*. The primary bacterial host for the *mcr-1* gene is *E. coli* (83.75%), followed by *Salmonella* as the second most common (8.57%), and *K. pneumoniae* ranking third (4.13%). In contrast, the *mcr-8* gene exhibits a distinct host preference, with its primary host strain being *K. pneumoniae* (92.68%), followed by *E. coli* (2.44%) [National Center for Biotechnology Information (NCBI), 2024]. This host-specific distribution pattern suggests that different *mcr* genes may disseminate within pathogen populations through distinct capacities for transmission.

A systematic analysis encompassing clinical strains from 2000 to 2020 confirmed that the detection rate of the *mcr-1* gene in global polymyxin-resistant *E. coli* reached as high as 97.89%, significantly exceeding other *mcr* subtypes (Mmatli et al., 2022). In contrast, the *mcr-8* gene shows a sporadic distribution in *E. coli* and has been reported in clinical and environmental isolates from countries including Thailand, Malaysia, and Nigeria (Khanawapee et al., 2021; Lemlem et al., 2023; Ngbede et al., 2020).

Conversely, in *K. pneumoniae*, the detection rate of the *mcr-8* gene is high (Ling et al., 2020). *mcr-8* was initially identified in a swine-derived *K. pneumoniae* strain in China (Wang, 2018). Epidemiological survey data further reveal a low prevalence of the *mcr-1* gene in *K. pneumoniae*: a study collecting 7,401 rectal swab samples from healthy individuals across 30 provincial-level administrative regions and municipalities in China reported an *mcr-1* positivity rate of 0.11% in *K. pneumoniae* (Lu et al., 2020).

The transmission and prevalence of *mcr* genes pose a significant threat to public health. Notably, epidemiological data indicate that the *mcr-1* gene has a markedly more prevalent in *E. coli* than in *K. pneumoniae*, whereas *mcr-8* shows the opposite distribution pattern. The scientific community has not yet fully elucidated the impact of this host-specific distribution on bacterial fitness, virulence, and transmission capacity.

Bacterial fitness is typically defined as the ability of a bacterial genotype to survive and reproduce in a specific environment. It is widely accepted that the acquisition of resistance genes often incurs a fitness cost (Andersson, 2010). Bacterial virulence, a key determinant of pathogenicity, encompasses toxin production and invasiveness—the latter including adhesion, invasion, dissemination, and the ability to resist or evade host immune defenses. Existing evidence suggests that the impact of resistance on virulence depends primarily on bacterial species, resistance mechanism, environmental factors, and host immunity (Gunn et al., 2000). Studies have shown that *mcr* genes not only confer polymyxin resistance but also impose a fitness cost on the host bacterium, thereby limiting the persistence of resistant strains within bacterial communities (Li et al., 2021; Yang et al., 2020).

Based on the above, we hypothesize that the marked difference in the prevalence of *mcr-1* and *mcr-8* between *E. coli* and *K. pneumoniae* stems from gene-specific effects on key bacterial properties, including fitness cost, conjugative transfer, plasmid stability, and virulence. Elucidating these host-gene specific relationships will advance our understanding of *mcr* gene dissemination and inform strategies to contain the spread of polymyxin-resistant pathogens.

## 2 Materials and methods

### 2.1 Bacterial strains and plasmids

*K.pneumoniae* ATCC 13883 and KP15255, and *E. coli* DH5α, ATCC 25922, and EC15255, were used as experimental strains in this study. The sources and resistance phenotypes of these strains are listed in Supplementary Table S1. The recombinant plasmid was introduced into *E. coli* DH5α by chemical transformation and into *K. pneumoniae* ATCC 13883 by electroporation. Transformants were selected on Luria–Bertani (LB) medium supplemented with 2 mg/L polymyxin and subsequently confirmed by PCR. The complete nucleotide sequences of *mcr-1* and *mcr-8* used in this study are provided in Supplementary material. Both genes were synthesized and cloned into the pCR2.1 vector backbone, and their integrity was confirmed by Sanger sequencing prior to all experiments.

### 2.2 Antimicrobial susceptibility testing

Antimicrobial susceptibility testing was performed following the recommendations of the Clinical and Laboratory Standards Institute (CLSI, M100-Ed32, 2022) and the European Committee on Antimicrobial Susceptibility Testing (EUCAST, Version 15.0, 2025). Briefly, fresh colonies were suspended in sterile saline and adjusted to a 0.5 McFarland turbidity standard, then diluted 1:20 in cation-adjusted Mueller–Hinton broth (CAMHB). Antimicrobial agents were serially diluted two-fold in 96-well microtiter plates, with a final volume of 100 μL per well. Plates were incubated at 35 ± 2 °C for 16–20 h. The minimum inhibitory concentration (MIC) was defined as the lowest concentration of antimicrobial agent that inhibited visible growth, with *E. coli* ATCC 25922 was used as the reference strain.

### 2.3 Plasmid stability

A single colony was grown overnight in antibiotic-containing LB broth at 37 °C with shaking. The culture was then diluted 1:100 into fresh antibiotic-containing LB and grown to mid-log phase OD600=0.5 ; this was designated as generation 0 (G0). After determining the initial plasmid retention rate by plating onto antibiotic-containing and antibiotic-free agar plates, the culture was diluted 1:1024 into antibiotic-free LB and grown to mid-log phase again (G10). This procedure was repeated until G50. Plasmid retention rate at each generation was calculated as the number of colonies on antibiotic-containing plates divided by the number on antibiotic-free plates (Kramer, 2016). All experiments were performed with three independent biological replicates, each conducted on separate days with freshly transformed colonies. For each biological replicate, three technical replicates were plated at each time point. Statistical comparisons of plasmid retention rates between transformant groups were performed using two-way analysis of variance (ANOVA). Statistical analyses were conducted using SPSS 27.0, and a *p*-value < 0.05 was considered statistically significant.

### 2.4 *In vitro* growth rate assay

The resuscitated and diluted strains were prepared into bacterial suspensions. The optical density (OD) of these suspensions was measured using a BioscreenC reader over a period of 20 h. Growth curves were generated from three independent biological replicates, each with three technical replicates per strain. The relative growth rate of the strains was calculated, and data analysis was performed using a non-parametric MannWhitney U-test, followed by Dunn’s multiple comparison test (Zwietering et al., 1990).

### 2.5 Bacterial conjugation assays

The plasmid conjugation efficiency of *mcr-1*-carrying *E. coli* and *K. pneumoniae* strains was investigated following a previously described method with minor modifications (Quan et al., 2023). Conjugation experiments were conducted using clinical strains *E. coli* EC15255 and *K. pneumoniae* KP15255, both harboring the *mcr-1* gene, as donor strains, with rifampicin-resistant *K. pneumoniae* ATCC 13883 (serving as recipient strain). Transconjugants were selected on LB agar supplemented with 2 mg/L polymyxin and 100 mg/L rifampicin. The presence of the *mcr-1* gene in the transconjugants was further confirmed by PCR. The conjugation frequency was calculated as the ratio of transconjugants over recipient cells. Conjugation assays were performed in three independent biological replicates, each with three technical replicates. Conjugation frequencies (transconjugants per recipient) were log10-transformed prior to statistical analysis to normalize distribution. Comparisons between donor groups were performed using the unpaired two-tailed Student’s *t*-test. A *p*-value < 0.05 was considered statistically significant.

### 2.6 *Galleria mellonella* larvae infection model

The *G. mellonella* larvae infection model was used to assess the *in vivo* virulence of the transformed strains. Upon arrival, larvae were stored in the dark and used within 3 days. Bacterial pellets were washed with sterile saline and diluted to the appropriate concentration. Using a microsyringe, 10 μL of the bacterial suspension was injected into the left anterior proleg of each larva; control larvae received 10 μL of PBS. After injection, larvae were incubated at 37 °C, and survival was recorded every 12 h for 3 days. Death was defined as the absence of response to touch. Survival data were analyzed using Kaplan–Meier survival curves (Williamson et al., 2014). Each experiment was performed with three independent biological replicates. For each independent experiment, each experimental group consisted of 10 larvae, and a total of 30 larvae were tested per strain across all replicates.

## 3 Reaults

### 3.1 Stability of *mcr-1*-carrying plasmids and *mcr-8*-carrying plasmids in *K. pneumoniae* and *E. coli*

**Figure 1:**
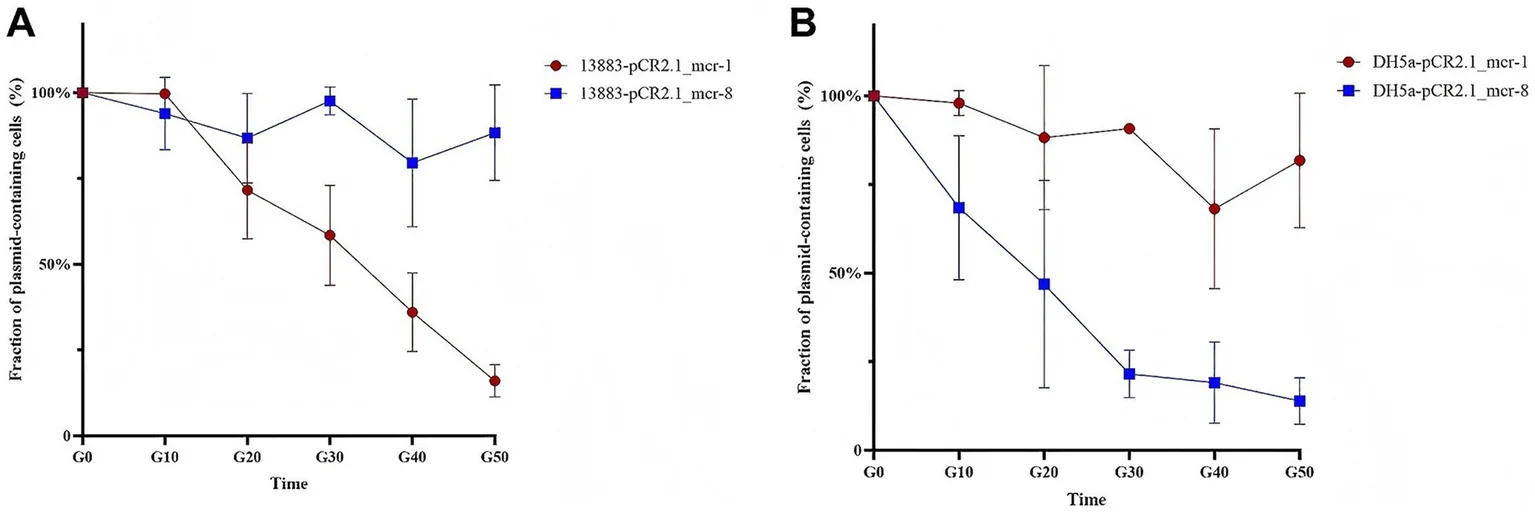
Stability of *mcr-1* and *mcr-8* plasmids in *K. pneumoniae* ATCC 13883 **(A)** and *E. coli* DH5α **(B)**. Data are presented as mean ± SD (*n* = 3). A two-way repeated measures ANOVA showed a significant group-by-time interaction (*p* = 0.0052 for **A**; *p* = 0.0152 for **B**). Statistical significance was set at *p* < 0.05.

The retention rates of *mcr* plasmids in the absence of selective pressure were evaluated through plasmid stability assays. The plasmids were introduced into *E. coli* DH5α and *K. pneumoniae* ATCC 13883 via chemical transformation and electroporation, respectively, and the resulting transformants were used for plasmid stability testing. As shown in Figure 1A, under conditions without selective pressure, the stability of the pCR2.1_mcr-1 plasmid in *K. pneumoniae* ATCC 13883 (strain 13,883-pCR2.1_mcr-1) began to decline from G10, with the retention rate dropping to a minimum of 16% by G50. In contrast, strain 13,883-pCR2.1_mcr-8 maintained a retention rate of 88.34% at G50, with its lowest point recorded as 79.54% at G40. Compared to the pCR2.1_mcr-1 plasmid, the pCR2.1_mcr-8 plasmid exhibited significantly better persistence in *K. pneumoniae*, and this difference was statistically significant (*p* = 0.0052). As shown in Figure 1B, in *E. coli* DH5α, the stability of the pCR2.1_mcr-8 plasmid began to decline from G10, reaching a minimum retention rate of 13.88% by G50. Conversely, strain DH5α-pCR2.1_mcr-1 showed a retention rate of 81.8% at G50, with its lowest point being 68.14% at G40. Compared to the pCR2.1_mcr-8 plasmid, the pCR2.1_mcr-1 plasmid demonstrated significantly better persistence in *E. coli*, and this difference was also statistically significant (*p* = 0.0152). Overall, a marked contrast in plasmid stability was observed: the *mcr-8* plasmid was significantly more stable in *K. pneumoniae*, while the *mcr-1* plasmid was significantly more persistent in *E. coli*.

### 3.2 Relative growth rates of *mcr-1* and *mcr-8* plasmids in in *K. pneumoniae* and *E. coli*

**Figure 2:**
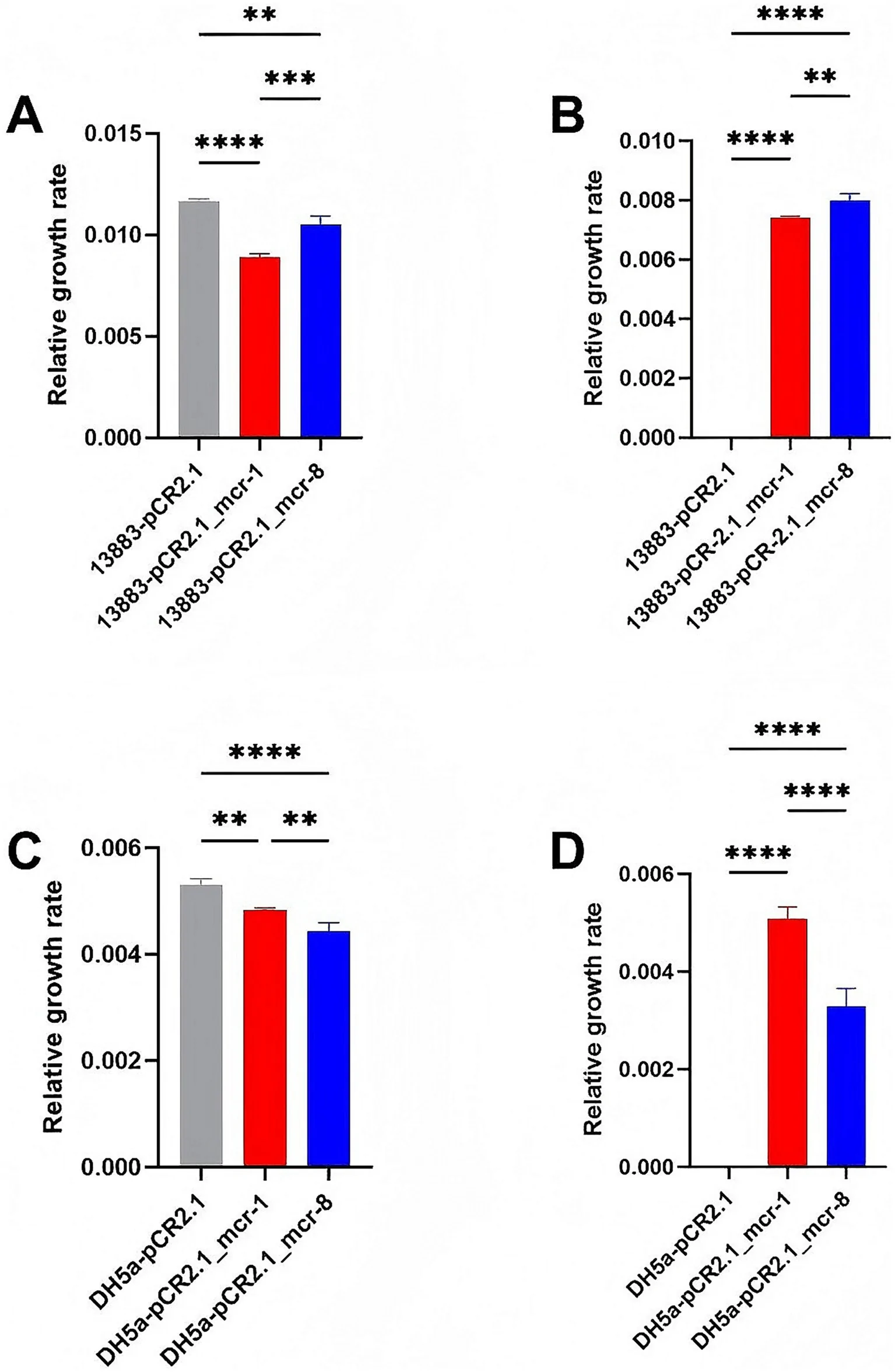
Relative growth rates of *K. pneumoniae* ATCC 13883 and *E. coli* DH5α harboring *mcr-1* or *mcr-8* plasmids in the absence and presence of polymyxin. **(A)** Relative growth rates of *K. pneumoniae* ATCC 13883 transformants (empty vector, pCR2.1_*mcr-1*, pCR2.1_*mcr-8*) in the absence of polymyxin. **(B)** Relative growth rates of *K. pneumoniae* transformants in the presence of polymyxin (1 mg/L). **(C)** Relative growth rates of *E. coli* DH5α transformants in the absence of polymyxin. **(D)** Relative growth rates of *E. coli* transformants in the presence of polymyxin (1 mg/L). For panels B and D, no growth was observed for the empty-vector control strains under polymyxin pressure throughout the monitoring period, and their relative growth rates were set to zero. Data are presented as mean ± SD (*n* = 3). Data were analyzed using one-way ANOVA followed by Tukey’s multiple comparison test. ****p < 0.0001, ***p < 0.001, **p < 0.01, *p < 0.05; ns, not significant.

The effects of pCR2.1_mcr-1 and pCR2.1_mcr-8 plasmids on the relative growth rates of *E. coli* and *K. pneumoniae* were evaluated under both in the absence and presence of polymyxin conditions using growth curve analysis. As shown in Figure 2A, in the absence of polymyxin pressure, the *mcr-8*-carrying strain exhibited a significantly higher relative growth rate than the *mcr-1*-carrying strain in *K. pneumoniae*. Under polymyxin pressure, this advantage was maintained, with the *mcr-8*-carrying strain still showing a higher relative growth rate in *K. pneumoniae* (Figure 2B). In contrast, as shown in Figure 2C, in the absence of polymyxin pressure, the *mcr-1*-carrying strain showed a superior relative growth rate in *E. coli*. This host-dependent growth advantage was also maintained under polymyxin pressure, where the *mcr-1*-carrying strain continued to exhibit a higher relative growth rate in *E. coli* (Figure 2D).

### 3.3 Efficiencies of transfer of *mcr-1*-carrying plasmids into *K. pneumoniae* and *E. coli*

Two clinical strains, EC15255 and KP15255, harboring an identical *mcr-1*-carrying plasmid, were included in this study. To investigate the horizontal transfer potential of this plasmid, conjugation experiments were performed, and the conjugation frequencies were determined.

In previous experiments (Quan et al., 2023), the transferability of the *mcr-1* plasmid from both donor strains to EC600 was assessed. The conjugation frequency from EC15255 to EC600 ranged from 2.33 × 10^−3^ to 6.49 × 10^−3^ transconjugants per recipient cell, while that from KP15255 to EC600 ranged from 1.59 × 10^−4^ to 2.33 × 10^−4^ transconjugants per recipient cell. No statistically significant difference was observed between the two donor strains in their conjugation efficiency to EC600 (*p* = 0.0582).

In the present study, further conjugation assays were conducted using ATCC 13883 as the recipient strain. The results showed that the conjugation frequency from EC15255 to ATCC 13883 ranged from 0.97 × 10^−4^ to 1.38 × 10^−4^ transconjugants per recipient cell, whereas that from KP15255 to ATCC 13883 ranged from 0.6 × 10^−5^ to 1.17 × 10^−5^ transconjugants per recipient cell. The difference in conjugation efficiency between the two donor strains was statistically significant (*p* < 0.0001).

### 3.4 *G. Mellonella* infection model of *mcr-1* and *mcr-8* plasmids in *K. pneumoniae* and *E. coli*

The *G. mellonella* larval infection model was used to evaluate the virulence of empty vector strains and transformants. Each group consisted of 15 larvae, which were injected with a standardized bacterial suspension. Larval survival was monitored to assess the impact of the *mcr-1* and *mcr-8* genes on bacterial virulence.

**Figure 3:**
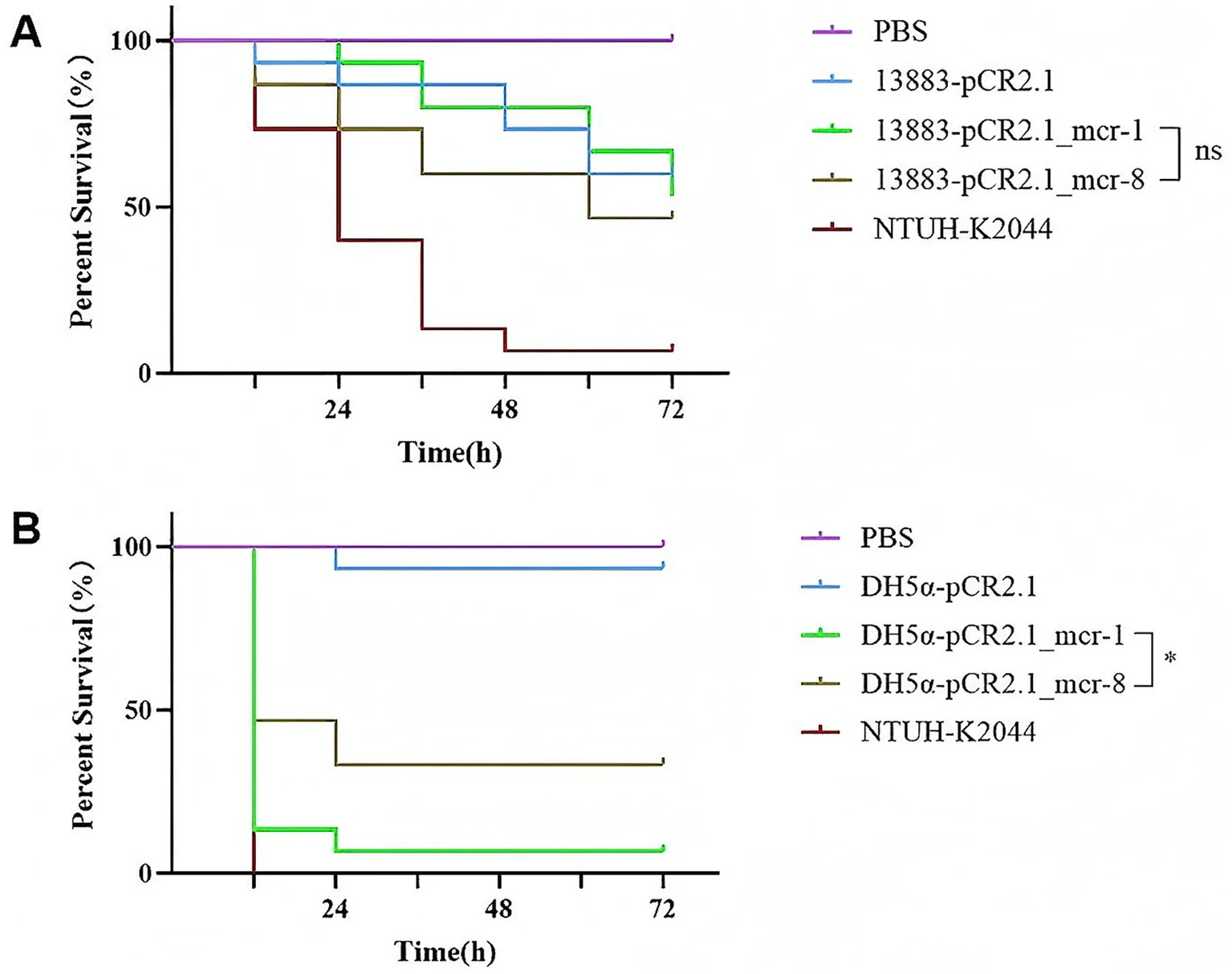
Virulence of *mcr-1* and *mcr-8* transformants in *G. mellonella* larvae. **(A)** Survival curves of *K. pneumoniae* ATCC 13883 transformants. Larvae were infected with empty vector strain (13883-pCR2.1), *mcr-1*-carrying transformant (13883-pCR2.1_mcr-1), or *mcr-8*-carrying transformant (13883-pCR2.1_mcr-8). PBS and NTUH-K2044 served as negative and positive controls, respectively. **(B)** Survival curves of *E. coli* DH5α transformants. Larvae were infected with empty vector strain (DH5α-pCR2.1), mcr-1-carrying transformant (DH5α-pCR2.1_mcr-1), or *mcr-8-*carrying transformant (DH5α-pCR2.1_mcr-8). PBS and NTUH-K2044 served as negative and positive controls, respectively. Larval survival was monitored for 72 h post-infection. Each group consisted of 15 larvae. Survival curves were plotted using the Kaplan–Meier method, and statistical significance was determined by the log-rank test.

As depicted in Figure 3A, in *K. pneumoniae* ATCC 13883, all larvae in the PBS control group survived, indicating a stable experimental system. The NTUH-K2044 positive control group exhibited a survival rate of only 7%, serving as a high-virulence reference. Larvae injected with the 13,883-pCR2.1 empty vector strain showed a 72-h survival rate of 60%, suggesting moderate pathogenicity. The 13,883-pCR2.1_mcr-1 transformant group had a 72-h survival rate of 53.33%, while the 13,883-pCR2.1_mcr-8 transformant group exhibited substantial mortality between 12 and 60 h post-injection, with a final survival rate of 46.67% at 72 h. Although the survival rate of the *mcr-8*-carrying transformant was lower than that of the *mcr-1*-carrying transformant, the difference was not statistically significant.

Figure 3B illustrates that in *E. coli* DH5α, all larvae in the PBS control group survived, confirming system stability. The NTUH-K2044 positive control group showed complete mortality, serving as a high-virulence control. Larvae injected with the DH5α-pCR2.1 empty vector strain displayed a 72-h survival rate of 93%, indicating low virulence. The DH5α-pCR2.1_mcr-1 transformant group exhibited a markedly reduced survival rate of 6.67% at 72 h, reflecting a rapid lethal effect. In contrast, the DH5α-pCR2.1_mcr-8 transformant group, although showing some early virulence, achieved a 72-h survival rate of 33.33%, which was significantly higher than that of the *mcr-1*-carrying transformant.

These findings demonstrate that the *E. coli* transformant harboring the *mcr-1* gene exhibited significantly enhanced virulence, with a 72-h survival rate of 6.67%, whereas the *mcr-8*-carrying *E. coli* transformant showed a survival rate of 33.33%, representing a statistically significant difference. In contrast, no significant difference in virulence was observed between the *mcr-1* and *mcr-8* genes in the *K. pneumoniae* transformants.

## 4 Discussion

The introduction of *mcr-1* and *mcr-8* altered bacterial growth characteristics compared to the empty vector control, and all transformants showed markedly increased polymyxin MICs. These observations align with the known function of MCR proteins as phosphoethanolamine transferases that modify lipid A (Zhu et al., 2021). The clinical strains EC15255 and KP15255 were both multidrug-resistant, which further suggests that the spread of *mcr* genes often goes hand in hand with other resistance determinants, adding to the overall public health burden (Wang et al., 2024; Zhang et al., 2022).

Our plasmid stability assay revealed that the stability of pCR2.1_mcr-1 and pCR2.1_mcr-8 was highly host-dependent. In the absence of polymyxin selective pressure, pCR2.1_mcr-8 exhibited markedly higher stability in *K. pneumoniae* than pCR2.1_mcr-1, whereas the opposite pattern was observed in *E. coli*, where pCR2.1_mcr-1 was retained more efficiently. These differences are consistent with the known concept of host-specific plasmid interactions and the fitness costs associated with resistance gene expression (Carroll and Wong, 2018; Wu et al., 2018). Host-plasmid interactions encompass processes such as plasmid replication, maintenance, and transfer, as well as reciprocal influences between the plasmid and the host genome. Such interactions can profoundly affect host metabolism, overall fitness, and evolutionary trajectories (Yano et al., 2016). Accordingly, the enhanced stability of pCR2.1_mcr-8 in *K. pneumoniae* could, in principle, reflect an efficient partitioning system or a reduced metabolic burden imposed by this plasmid. Conversely, pCR2.1_mcr-1 may achieve superior stability in *E. coli* through analogous mechanisms. It is plausible that the level of *mcr* gene expression also contributes to these host-dependent differences. Higher expression of *mcr-8* might, hypothetically, be better tolerated in *K. pneumoniae*, whereas the expression pattern of *mcr-1* might be more compatible with the physiological environment of *E. coli* (Li et al., 2021; Nang et al., 2018). These stability differences lhave significant implications for the dissemination of resistance genes. Specifically, the high retention of *mcr-8* in *K. pneumoniae* may facilitate the long-term persistence of polymyxin resistance, while the stability of *mcr-1* in *E. coli* could render this species a potential reservoir for further horizontal gene transfer.

We observed notable host-specific differences in bacterial fitness. The *K. pneumoniae* transformant harboring pCR2.1_mcr-8 exhibited enhanced growth both in the presence and absence of polymyxin selective pressure, whereas the *E. coli* transformant carrying pCR2.1_mcr-1 showed superior fitness under both conditions. A possible explanation for these differences is that they may reflect variations in gene expression regulation across hosts and the compatibility of the plasmid with host metabolic systems. For instance, in *Pseudomonas aeruginosa*, the ParRS and CprRS two-component systems specifically activate the *arnBCADTEF* operon upon sensing polymyxins, leading to 4-aminoarabinose modification of lipid A (Li et al., 2023). Therefore, by analogy, it is tempting to speculate that *K. pneumoniae* harbors specific, as-yet-unidentified regulatory factors that more efficiently drive *mcr-8* transcription, thereby enhancing plasmid stability, reducing energy costs, and promoting growth. In contrast, *E. coli* may lack these compatible regulatory elements, resulting in suboptimal *mcr-8* expression and diminished fitness benefits. For *E. coli*, pCR2.1_mcr-1 may employ compensatory mechanisms, such as modulating plasmid copy number, to enhance fitness (Ogunlana et al., 2023). It should also be noted that in the present study, transformants carrying the empty vector consistently exhibited greater fitness than those harboring *mcr* genes. This observation is consistent with previous reports demonstrating that expression of *mcr* genes imposes a significant fitness cost, leading to reduced growth rates, compromised membrane integrity, and increased bacterial mortality (Yang et al., 2017).

Our conjugation experiments demonstrated that the *mcr-1* gene could be transferred to recipient strains. However, the transfer efficiency varied markedly depending on the donor. Specifically, when *E. coli* EC15255 served as the donor, the *mcr-1* plasmid transferred to *K. pneumoniae* ATCC 13883 at significantly higher frequency than when *K. pneumoniae* KP15255 was used as the donor, despite both strains harboring the same *mcr-1* plasmid. Several non-exclusive factors may account for this observation, although their specific contributions remain to be determined. One possibility involves host restriction-modification systems. Another possibility is that the expression of conjugation-related genes, such as the type IV secretion system genes *virB* or *traC*, may differ between the two species (Liu et al., 2023). Furthermore, environmental factors such as polymyxin have been reported to enhance the activity of the secretion system, increase membrane permeability, and promote pilus production, all of which can elevate conjugation frequency (Xiao et al., 2022). We also observed that the transconjugants exhibited lower polymyxin MICs than the original donors. This could be attributed to the intrinsic physiology of the recipient strain, lower expression of the resistance gene, or reduced plasmid stability in the new host (Samantha and Vrielink, 2020). It has previously been shown that the same plasmid does not always confer identical resistance levels across different bacterial hosts (Dunn et al., 2021). Moreover, the fitness cost associated with *mcr-1* may further influence plasmid maintenance and the strength of resistance phenotype expression in the recipient.

In the *G. mellonella* infection model, the effect of *mcr-1* and *mcr-8* on bacterial virulence was highly dependent on the host background. For *K. pneumoniae* transformants, both *mcr-1* and *mcr-8* exhibited slightly higher virulence compared to the empty vector control. However, the difference was not statistically significant. This may be attributed to the intrinsically high virulence of *K. pneumoniae*, which could mask any additional effect conferred by exogenous *mcr* genes (Ah et al., 2014; McConville et al., 2020). Studies have shown that *K. pneumoniae* virulence often relies on capsule synthesis mutations, which help the bacterium evade phagocytosis or form biofilms depending on the infection type (Fu et al., 2025; Mondol et al., 2025). Notably, larvae infected with the *mcr-8*-positive strain predominantly died between 12 and 60 h post-infection. This observation may reflect accelerated early virulence expression, although it did not significantly alter overall lethality. So maybe the impact of *mcr-8* on *K. pneumoniae* virulence is limited to certain infection stages or specific immune pathways. In contrast, the *E. coli* transformant carrying *mcr-1* exhibited a rapid lethal effect, and its virulence was markedly higher than that of the *mcr-8*-carrying transformant. One plausible interpretation, though requiring further validation, is that this difference may be explained by the fact that *E. coli* DH5α is a low-virulence cloning host. It has been genetically modified and lacks the typical virulence factors found in wild-type strains (Monk et al., 2016). Consequently, the lipid A modification mediated by *mcr-1* could, in principle, be more likely to enhance endotoxin activity or interfere with host immune recognition (Jiang et al., 2020). It is important to note, however, that the *G. mellonella* model, while widely used as an *in vivo* surrogate, does not fully recapitulate mammalian infections, particularly regarding adaptive immunity and tissue-specific barriers. Therefore, the virulence differences observed here should be interpreted primarily as host-dependent phenotypic effects under controlled laboratory conditions, and direct extrapolation to clinical outcomes should be made with caution. Future studies using mammalian infection models will be essential to assess the true clinical relevance of these findings.

It should be noted that the *mcr-1* and *mcr-8* genes used in this study are of comparable size and were inserted into the same pCR2.1 vector backbone, ensuring that the total genetic cargo was standardized between the two constructs. This allows us to exclude cargo size and genetic context as confounding variables when interpreting the observed host-dependent differences. Nevertheless, we acknowledge that the larger genome of *K. pneumoniae* may contribute to its greater accommodation of *mcr-8* carriage, consistent with previous observations that bacteria with larger genomes can harbor excess genetic cargo with reduced fitness costs (Projan, 2007; Vanacker et al., 2023). Codon usage bias may also contribute to the differential fitness effects of *mcr-1* and *mcr-8* observed between *E. coli* and *K. pneumoniae*. Recent studies have demonstrated that the acquisition and maintenance of mcr genes are partly dependent on codon optimization, with the 5′-end codon optimality serving as a critical regulator of mRNA stability, protein expression, and fitness cost. Notably, genes with a higher proportion of non-optimal codons at the 5′ end tend to exhibit reduced mRNA stability and lower protein expression, thereby minimizing disruption to membrane integrity and imposing a lower fitness cost on the bacterial host. (Liang et al., 2025; Song, 2024) Within this framework, it is plausible that the differential distribution of codon optimality at the 5′ end of *mcr-1* and *mcr-8* may partly account for their host-specific fitness patterns observed in our study. However, this interpretation remains speculative in the absence of direct experimental validation, and future studies—including mRNA stability assays, protein expression quantification, and targeted synonymous codon substitution—are warranted to systematically evaluate the contribution of 5′-end codon optimality to the host-dependent behavior of *mcr* genes.

Several limitations of this study should be acknowledged. First, the recombinant plasmids pCR2.1_mcr-1 and pCR2.1_mcr-8 used herein may not fully recapitulate the complex genetic contexts of naturally occurring *mcr*-bearing plasmids. Native plasmids typically harbor additional cargo genes, insertion sequences, and regulatory elements that can modulate fitness, stability, and horizontal transfer, and their absence from our constructs may have influenced the observed phenotypes. Second, the laboratory strains *E. coli* DH5α and *K. pneumoniae* ATCC 13883, along with the clinical isolates EC15255 and KP15255, may not adequately capture the genetic diversity of clinical populations. This is particularly relevant for *E. coli* DH5α, a genetically modified cloning host that lacks native virulence determinants and may engage in plasmid–host interactions that differ substantially from those of wild-type clinical strains. Third, our use of only one clinical isolate per bacterial species limits the generalizability of our conclusions, as strain-to-strain variability within each species could affect the host-dependent patterns observed here. Consequently, extrapolation of our findings to clinical settings warrants caution, and future investigations employing a broader panel of clinical *mcr*-positive isolates—preferably carrying their native plasmids—will be essential to validate and extend our results. Fourth, our conjugation experiments focused exclusively on *mcr-1*, as we were unable to successfully transfer *mcr-8*-carrying plasmids under the same experimental conditions. In preliminary experiments using a clinical strain (15919) that harbors both *mcr-1* and *mcr-8* on distinct plasmids, we attempted to transfer both resistance determinants to the rifampicin-resistant *K. pneumoniae* ATCC 13883 recipient under various conditions (e.g., temperature, mating time, and donor-to-recipient ratios). However, no transconjugants could be recovered on double-antibiotic selective plates despite multiple independent attempts. The precise reasons for this technical difficulty remain unclear but may involve the larger genetic load of the *mcr-8*-bearing plasmid, host restriction-modification barriers, or reduced plasmid stability in the donor strain under conjugation conditions. Consequently, we are unable to compare the transfer potential of *mcr-8* with that of *mcr-1*, and this represents a notable limitation of the present study. Future work employing alternative conjugation systems (e.g., filter mating or liquid mating with optimized conditions) or native *mcr-8*-bearing plasmids from clinical isolates will be necessary to address this question.

## 5 Conclusion

Through systematic investigation, this study elucidated the host-dependent transmission and adaptation patterns of *mcr-1* and *mcr-8*. The fitness effects of these genes are closely associated with host genetic backgrounds: *mcr-1* confers higher plasmid stability and fitness advantages in *E. coli*, while *mcr-8* does so in *K. pneumoniae*. Additionally, *mcr-1* demonstrated significantly higher conjugation efficiency and enhanced virulence when carried by *E. coli*. Based on these host-specific transmission dynamics and fitness differences, we recommend strengthening resistance surveillance networks with focused screening for *mcr* genes in high-risk hosts, and developing differentiated prevention strategies for distinct bacterial hosts to curb cross-species resistance gene spread and mitigate hospital-acquired outbreak risks.

## Data Availability

The original contributions presented in the study are included in the article/Supplementary material, further inquiries can be directed to the corresponding authors.

## Footnotes

1
https://www.chinets.com

## References

[ref1] Aghapour Z. Gholizadeh P. Ganbarov K. Bialvaei A. Z. Mahmood S. S. Tanomand A. . (2019). Molecular mechanisms related to colistin resistance in Enterobacteriaceae. IDR 12, 965–975. doi: 10.2147/IDR.S199844, 31190901 PMC6519339

[ref2] Ah Y.-M. Kim A.-J. Lee J.-Y. (2014). Colistin resistance in *Klebsiella pneumoniae*. Int. J. Antimicrob. Agents 44, 8–15. doi: 10.1016/j.ijantimicag.2014.02.016, 24794735

[ref3] Andersson D. I. (2010). Antibiotic resistance and its cost: is it possible to reverse resistance? Nat. Rev. Microbiol. 8, 260–271. doi: 10.1038/nrmicro2319, 20208551

[ref4] Carroll A. C. Wong A. (2018). Plasmid persistence: costs, benefits, and the plasmid paradox. Can. J. Microbiol. 64, 293–304. doi: 10.1139/cjm-2017-0609, 29562144

[ref5] Dunn S. Carrilero L. Brockhurst M. McNally A. (2021). Limited and strain-specific transcriptional and growth responses to acquisition of a multidrug resistance plasmid in genetically diverse *Escherichia coli* lineages. mSystems 6:e00083. doi: 10.1128/mSystems.00083-21 33906912 PMC8092126

[ref6] Fu Y. Yin M. Cao L. Lu Y. Li Y. Zhang L. (2025). Capsule mutations serve as a key strategy of phage resistance evolution of K54 hypervirulent *Klebsiella pneumoniae*. Commun. Biol. 8:257. doi: 10.1038/s42003-025-07687-8, 39966630 PMC11836320

[ref7] Gunn J. S. Ryan S. S. Van Velkinburgh J. C. Ernst R. K. Miller S. I. (2000). Genetic and functional analysis of a PmrA-PmrB-regulated locus necessary for lipopolysaccharide modification, antimicrobial peptide resistance, and Oral virulence of *Salmonella enterica* serovar typhimurium. Infect. Immun. 68, 6139–6146. doi: 10.1128/IAI.68.11.6139-6146.2000, 11035717 PMC97691

[ref8] Jiang B. Du P. Jia P. Liu E. Kudinha T. Zhang H. . (2020). Antimicrobial susceptibility and virulence of mcr-1-positive Enterobacteriaceae in China, a Multicenter longitudinal epidemiological study. Front. Microbiol. 11:1611. doi: 10.3389/fmicb.2020.01611, 32849334 PMC7399235

[ref9] Khanawapee A. Kerdsin A. Chopjitt P. Boueroy P. Hatrongjit R. Akeda Y. . (2021). Distribution and molecular characterization of *Escherichia coli* Harboring *mcr* genes isolated from slaughtered pigs in Thailand. Microb. Drug Resist. 27, 971–979. doi: 10.1089/mdr.2020.0242, 33325796

[ref10] Kramer M. G. (2016). Determination of plasmid Segregational stability in a growing bacterial population. Methods Mol Biol 1409, 125–133. doi: 10.1007/978-1-4939-3515-4_11 26846807

[ref11] Lemlem M. Aklilu E. Mohamed M. Kamaruzzaman N. F. Zakaria Z. Harun A. . (2023). Phenotypic and genotypic characterization of colistin-resistant *Escherichia Coli* with mcr-4, mcr-5, mcr-6, and mcr-9 genes from broiler chicken and farm environment. BMC Microbiol. 23:392. doi: 10.1186/s12866-023-03118-y, 38062398 PMC10704802

[ref12] Li W. Liu Z. Yin W. Yang L. Qiao L. Song S. . (2021). MCR expression conferring varied fitness costs on host Bacteria and affecting Bacteria virulence. Antibiotics 10:872. doi: 10.3390/antibiotics10070872, 34356793 PMC8300855

[ref13] Li L. Ma J. Cheng P. Li M. Yu Z. Song X. . (2023). Roles of two-component regulatory systems in *Klebsiella pneumoniae*: regulation of virulence, antibiotic resistance, and stress responses. Microbiol. Res. 272:127374. doi: 10.1016/j.micres.2023.127374, 37031567

[ref14] Liang L. Li Y. Wang L. Wang W. Zhang Y. Zhao H. . (2025). Fitness costs of mobilised colistin resistance gene 3 (mcr-3): systematic review, epidemiological study, and functional analysis. EBioMedicine 120:105923. doi: 10.1016/j.ebiom.2025.105923, 40945051 PMC12571581

[ref15] Ling Z. Yin W. Shen Z. Wang Y. Shen J. Walsh T. R. (2020). Epidemiology of mobile colistin resistance genes mcr-1 to mcr-9. J. Antimicrob. Chemother. 75, 3087–3095. doi: 10.1093/jac/dkaa205, 32514524

[ref16] Liu J.-H. Liu Y.-Y. Shen Y.-B. Yang J. Walsh T. R. Wang Y. . (2024). Plasmid-mediated colistin-resistance genes: mcr. Trends Microbiol. 32, 365–378. doi: 10.1016/j.tim.2023.10.006, 38008597

[ref17] Liu Y.-Y. Wang Y. Walsh T. R. Yi L.-X. Zhang R. Spencer J. . (2016). Emergence of plasmid-mediated colistin resistance mechanism MCR-1 in animals and human beings in China: a microbiological and molecular biological study. Lancet Infect. Dis. 16, 161–168. doi: 10.1016/S1473-3099(15)00424-7, 26603172

[ref18] Liu Y.-Y. Zhu X.-Q. Nang S. C. Xun H. Lv L. Yang J. . (2023). Greater invasion and persistence of *mcr-1* bearing plasmids in *Escherichia coli* than in *Klebsiella pneumoniae*. Microbiol. Spectrum 11, e03223–e03222. doi: 10.1128/spectrum.03223-22, 36975832 PMC10100767

[ref19] Lu J. Dong N. Liu C. Zeng Y. Sun Q. Zhou H. . (2020). Prevalence and molecular epidemiology of mcr-1-positive *Klebsiella pneumoniae* in healthy adults from China. J. Antimicrob. Chemother. 75, 2485–2494. doi: 10.1093/jac/dkaa210, 32516364

[ref20] McConville T. H. Annavajhala M. K. Giddins M. J. Macesic N. Herrera C. M. Rozenberg F. D. . (2020). CrrB positively regulates high-level polymyxin resistance and virulence in *Klebsiella pneumoniae*. Cell Rep. 33:108313. doi: 10.1016/j.celrep.2020.108313, 33113377 PMC7656232

[ref21] Mmatli M. Mbelle N. M. Osei Sekyere J. (2022). Global epidemiology, genetic environment, risk factors and therapeutic prospects of mcr genes: a current and emerging update. Front. Cell. Infect. Microbiol. 12:941358. doi: 10.3389/fcimb.2022.94135 36093193 PMC9462459

[ref22] Mondol S. M. Hossain M. A. Haque F. K. M. (2025). Comprehensive genomic insights into a highly pathogenic clone ST656 of mcr8.1 containing multidrug-resistant *Klebsiella pneumoniae* from Bangladesh. Sci. Rep. 15:5909. doi: 10.1038/s41598-025-90414-4, 39966674 PMC11836182

[ref23] Monk J. M. Koza A. Campodonico M. A. Machado D. Seoane J. M. Palsson B. O. . (2016). Multi-omics quantification of species variation of *Escherichia coli* links molecular features with strain phenotypes. Cell Systems 3, 238–251.e12. doi: 10.1016/j.cels.2016.08.013, 27667363 PMC5058344

[ref24] Nang S. C. Morris F. C. McDonald M. J. Han M.-L. Wang J. Strugnell R. A. . (2018). Fitness cost of mcr-1-mediated polymyxin resistance in *Klebsiella pneumoniae*. J. Antimicrob. Chemother. 73, 1604–1610. doi: 10.1093/jac/dky061, 29514208 PMC6693033

[ref25] National Center for Biotechnology Information (NCBI) (2024) National Database of Antibiotic Resistant Organisms (NDARO). Available online at: https://www.ncbi.nlm.nih.gov/pathogens/antimicrobial-resistance/ (Accessed Nov 22 2024).

[ref26] Ngbede E. O. Poudel A. Kalalah A. Yang Y. Adekanmbi F. Adikwu A. A. . (2020). Identification of mobile colistin resistance genes (mcr-1.1, mcr-5 and mcr-8.1) in Enterobacteriaceae and *Alcaligenes faecalis* of human and animal origin, Nigeria. Int. J. Antimicrob. Agents 56:106108. doi: 10.1016/j.ijantimicag.2020.106108, 32721596

[ref27] Ogunlana L. Kaur D. Shaw L. P. Jangir P. Walsh T. Uphoff S. . (2023). Regulatory fine-tuning of *mcr-1* increases bacterial fitness and stabilises antibiotic resistance in agricultural settings. ISME J. 17, 2058–2069. doi: 10.1038/s41396-023-01509-7, 37723338 PMC10579358

[ref28] Projan S. J. (2007). (Genome) size matters. Antimicrob. Agents Chemother. 51, 1133–1134. doi: 10.1128/AAC.01370-06, 17296743 PMC1855458

[ref29] Quan J. Hu H. Zhang H. Meng Y. Liao W. Zhou J. . (2023). Investigating possible interspecies communication of plasmids associated with transfer of third-generation cephalosporin, quinolone, and colistin resistance between simultaneously isolated Escherichia Coli and *Klebsiella Pneumoniae*. Microbiol. Spectrum 11, e0355422–e0355422. doi: 10.1128/spectrum.03554-22, 37125932 PMC10269620

[ref30] Samantha A. Vrielink A. (2020). Lipid a Phosphoethanolamine transferase: regulation, structure and immune response. J. Mol. Biol. 432, 5184–5196. doi: 10.1016/j.jmb.2020.04.022, 32353363

[ref31] Sati H. Carrara E. Savoldi A. Hansen P. Garlasco J. Campagnaro E. . (2025). The WHO bacterial priority pathogens list 2024: a prioritisation study to guide research, development, and public health strategies against antimicrobial resistance. Lancet Infect. Dis. 25, 1033–1043. doi: 10.1016/S1473-3099(25)00118-5, 40245910 PMC12367593

[ref32] Song K. (2024). Decoding the origins, spread, and global risks of *mcr-9* gene. EBioMedicine 108:105326. doi: 10.1016/j.ebiom.2024.105326, 39260038 PMC11416231

[ref33] Vanacker M. Lenuzza N. Rasigade J.-P. (2023). The fitness cost of horizontally transferred and mutational antimicrobial resistance in *Escherichia coli*. Front. Microbiol. 14:1186920. doi: 10.3389/fmicb.2023.1186920, 37455716 PMC10348881

[ref34] Wang X. (2018). Emergence of a novel mobile colistin resistance gene, *mcr-8*, in NDM-producing *Klebsiella pneumoniae*. Emerg. Microbes Infect. 7:122. doi: 10.1038/s41426-018-0124-z, 29970891 PMC6030107

[ref35] Wang Q. Wang W. Zhu Q. Shoaib M. Chengye W. Zhu Z. . (2024). The prevalent dynamic and genetic characterization of mcr-1 encoding multidrug resistant *Escherichia coli* strains recovered from poultry in Hebei, China. J. Global Antimicrobial Resistance 38, 354–362. doi: 10.1016/j.jgar.2024.04.001, 38795771

[ref36] Williamson D. A. Mills G. Johnson J. R. Porter S. Wiles S. (2014). In vivo correlates of molecularly inferred virulence among extraintestinal pathogenic *Escherichia coli* (ExPEC) in the wax moth *galleria mellonella* model system. Virulence 5, 388–393. doi: 10.4161/viru.27912, 24518442 PMC3979865

[ref37] Wu R. Yi L. Yu L. Wang J. Liu Y. Chen X. . (2018). Fitness advantage of mcr-1–bearing IncI2 and IncX4 plasmids in vitro. Front. Microbiol. 9:331. doi: 10.3389/fmicb.2018.00331, 29535696 PMC5835064

[ref38] Xiao X. Zeng F. Li R. Liu Y. Wang Z. (2022). Subinhibitory concentration of colistin promotes the conjugation frequencies of *Mcr-1* and *bla*_NDM-5_-positive plasmids. Microbiol. Spectr. 10:e02160-21. doi: 10.1128/spectrum.02160-21 PMC9045390 35230128

[ref39] Yang Q. Li M. Spiller O. B. Andrey D. O. Hinchliffe P. Li H. . (2017). Balancing mcr-1 expression and bacterial survival is a delicate equilibrium between essential cellular defence mechanisms. Nat. Commun. 8:2054. doi: 10.1038/s41467-017-02149-0, 29233990 PMC5727292

[ref40] Yang Q. E. MacLean C. Papkou A. Pritchard M. Powell L. Thomas D. . (2020). Compensatory mutations modulate the competitiveness and dynamics of plasmid-mediated colistin resistance in *Escherichia coli* clones. ISME J. 14, 861–865. doi: 10.1038/s41396-019-0578-6, 31896787 PMC7031280

[ref41] Yano H. Wegrzyn K. Loftie-Eaton W. Johnson J. Deckert G. E. Rogers L. M. . (2016). Evolved plasmid-host interactions reduce plasmid interference cost. Mol. Microbiol. 101, 743–756. doi: 10.1111/mmi.13407, 27121483 PMC5024541

[ref42] Zhang W. Lu X. Chen S. Liu Y. Peng D. Wang Z. . (2022). Molecular epidemiology and population genomics of tet(X4), blaNDM or mcr-1 positive *Escherichia coli* from migratory birds in southeast coast of China. Ecotoxicol. Environ. Saf. 244:114032. doi: 10.1016/j.ecoenv.2022.114032, 36084501

[ref43] Zhu X.-Q. Liu Y.-Y. Wu R. Xun H. Sun J. Li J. . (2021). Impact of mcr-1 on the development of high level colistin resistance in Klebsiella pneumoniae and *Escherichia coli*. Front. Microbiol. 12:666782. doi: 10.3389/fmicb.2021.666782, 33981294 PMC8108134

[ref44] Zwietering M. H. Jongenburger I. Rombouts F. M. van 't Riet K. (1990). Modeling of the bacterial growth curve. Appl. Environ. Microbiol. 56, 1875–1881. doi: 10.1128/aem.56.6.1875-1881.1990 16348228 PMC184525

