# Supplementary material for: Host-dependent effects of mcr-1 and mcr-8 on fitness, virulence, and transmission in Escherichia coli and Klebsiella pneumoniae
Source: Front Microbiol. 2026 Aug 12;17:1908233. doi: 10.3389/fmicb.2026.1908233 (PMC13506780; doi:10.3389/fmicb.2026.1908233)
Supplement: Supplementary file 1 [file Table_1.DOCX]

Supplementary Material

# 1 Table S1 **Characteristics of *Klebsiella pneumoniae* (KP) and *Escherichia coli* (*E. coli*) strains and their transformants/transconjugants used in this study**

| **Strains** | **Species** | **CST (mg/L)** | **PB (mg/L)** | **Resistance pattern^a^** |
| --- | --- | --- | --- | --- |
| 13883-pCR2.1 | KP | 0.5 | 0.25 | SCF, PTZ |
| 13883-pCR2.1_mcr-1 | KP | ＞32 | ＞32 | SCF, PTZ |
| 13883-pCR2.1_mcr-8 | KP | 8 | 4 | SCF, PTZ |
| DH5α-pCR2.1 | *E. coli* | 0.03 | 0.03 | - |
| DH5α-pCR2.1_mcr-1 | *E. coli* | 16 | 16 | - |
| DH5α-pCR2.1_mcr-8 | *E. coli* | 4 | 4 | - |
| EC15255 | *E. coli* | 16 | 16 | SCF, LEV, TET |
| KP15255 | KP | ＞32 | ＞32 | SCF, LEV, TCG, MIN, TET |
| EC15255C^b^ | *E. coli* | 8 | 8 | SCF, LEV |
| KP15255C^c^ | KP | 16 | 16 | SCF, LEV |
| ATCC 25922^d^ | *E. coli* | 0.12 | 0.25 | - |

^a^Antimicrobial susceptibility was determined and evaluated according to CLSI (https://clsi.org/standards/products/microbiology/,M100-Ed32,2022) and EUCAST (https://www.eucast.org/bacteria/clinical-breakpoints-and-interpretation/clinical-breakpoint-tables/,Version 15.0, 2025). CST, Colistin (Polymyxin E); PB, Polymyxin B; CAZ, Ceftazidime; FEP, Cefepime; SCF, Cefoperazone/Sulbactam; PTZ, Piperacillin/Tazobactam; CZA, Ceftazidime/Avibactam; ETP, Ertapenem; MEM, Meropenem; LEV, Levofloxacin; AMK, Amikacin; TGC, Tigecycline; MIN, Minocycline; TET, Tetracycline.

^b^ Transconjugant of *E. coli* isolate EC15255.

^c^ Transconjugant of *K. pneumoniae* isolate KP15255.

^d^ Quality control for antimicrobial susceptibility testing.

# 2 Table S2 **Primers used in this study**

| **P****rimer** | **Sequence (5’ to 3’)** | **Gene** |
| --- | --- | --- |
| mcr-1_F | ATTGCCGCAATTATCCCACC | *mcr-1* |
| mcr-1_R | GCAAACTTGGTAGCACACCC |  |
| mcr-8_F | AACCGCCAGAGCACAGAATT | *mcr-8* |
| mcr-8_R | TTCCCCCAGCGATTCTCCAT |  |
| pgi_F | TAACTTCGCTGCCGTCTTTC | *pgi* |
| pgi_R | GATCGGCATCTGGTACAACA |  |
| gapA_F | TTGTTGCTGAAGCAACCGGT | *gapA* |
| gapA_R | AACAACAGATACGTTCGGAG |  |

# 3 Table S3 Sequence information of *mcr-1* and *mcr-8*

| Gene | Sequence (5’ to 3’) |
| --- | --- |
| *mcr-1* | atgatgcagcatacttctgtgtggtaccgacgctcggtcagtccgtttgttcttgtggcgagtgttgccgttttcttgaccgcgaccgccaatcttaccttttttgataaaatcagccaaacctatcccatcgcggacaatctcggctttgtgctgacgatcgctgtcgtgctctttggcgcgatgctactgatcaccacgctgttatcatcgtatcgctatgtgctaaagcctgtgttgattttgctattaatcatgggcgcggtgaccagttattttactgacacttatggcacggtctatgatacgaccatgctccaaaatgccctacagaccgaccaagccgagaccaaggatctattaaacgcagcgtttatcatgcgtatcattggtttgggtgtgctaccaagtttgcttgtggcttttgttaaggtggattatccgacttggggcaagggtttgatgcgccgattgggcttgatcgtggcaagtcttgcgctgattttactgcctgtggtggcgttcagcagtcattatgccagtttctttcgcgtgcataagccgctgcgtagctatgtcaatccgatcatgccaatctactcggtgggtaagcttgccagtattgagtataaaaaagccagtgcgccaaaagataccatttatcacgccaaagacgcggtacaagcaaccaagcctgatatgcgtaagccacgcctagtggtgttcgtcgtcggtgagacggcacgcgccgatcatgtcagcttcaatggctatgagcgcgatactttcccacagcttgccaagatcgatggcgtgaccaattttagcaatgtcacatcgtgcggcacatcgacggcgtattctgtgccgtgtatgttcagctatctgggcgcggatgagtatgatgtcgataccgccaaataccaagaaaatgtgctggatacgctggatcgcttgggcgtaagtatcttgtggcgtgataataattcggactcaaaaggcgtgatggataagctgccaaaagcgcaatttgccgattataaatccgcgaccaacaacgccatctgcaacaccaatccttataacgaatgccgcgatgtcggtatgctcgttggcttagatgactttgtcgctgccaataacggcaaagatatgctgatcatgctgcaccaaatgggcaatcacgggcctgcgtattttaagcgatatgatgaaaagtttgccaaattcacgccagtgtgtgaaggtaatgagcttgccaagtgcgaacatcagtccttgatcaatgcttatgacaatgccttgcttgccaccgatgatttcatcgctcaaagtatccagtggctgcagacgcacagcaatgcctatgatgtctcaatgctgtatgtcagcgatcatggcgaaagtctgggtgagaacggtgtctatctacatggtatgccaaatgcctttgcaccaaaagaacagcgcagtgtgcctgcatttttctggacggataagcaaactggcatcacgccaatggcaaccgataccgtcctgacccatgacgcgatcacgccgacattattaaagctgtttgatgtcaccgcggacaaagtcaaagaccgcaccgcattcatccgctga |
| *mcr-8* | atgttcaagtatcttttatctttcaaactgaacccggtacaacggacctgggctgcagcattttttttcactacaatcggcaacatagcactttggcaaacactatggattaatgtagatgttcataatatacataatctacttttttttgccagtctgccaatatttcttttctgctttctaagtatcttacttacaccagtcatggttattccatatttatgcaggcctctacttgtagttcttattctaatcagtgcctgctgtagttatttcatgatgaaatacaacatattaattgaccgcagcatggtgcaaaacttttttgagactaatcaggctgaattaacatcatacttatccgttccttttctttccactctatttctacttggcattgtaccagcaattatcctggcgttgccttcaacagacaataagcggggagcttttagaattgaattgtggtggttggcgcatatttgcatagctgtagtcttattagccatggttaccatggtgttttataaggattacgcatctctcatacgaaacaatatgcagattaaagaccaggctttaccttttaactttgtgcgtaatacgaatggttaccttaaaagaaaataccaggcatcttcaacaattctacaaagcgtgggggaggatgctgtacgtccaatatattcaaatgctccaccgaaactggtggttgtcgtcgtgggcgaaaccgccagagcacagaatttccagctgaatggctattcgcgggtaaccaacccctatctttccagacgacatgatgttatcagtttcaaaaatgtgtcgtcatgcggaacggctaccgcaatatcactaccctgcatgttctcgcgaatgtcacgtaacgaatacaatgaagtccgtgccgcatcagaagaaaacttgctggatatccttaaacgtacaggtgttgaggtgctatggcgcaacaataacaatggtggttgtaagggaatctgcaagcgagtacccacagatgatatgccggcaatgaaagtaattggggaatgtgttaacaaagatggtacatgctttgatgaggtgttattaaatcaactctcatcccgaattaatgcaatgcagggtgatgcgcttattgttttacatcaaatgggcagtcatggaccaacatattttgaacgttatccgtctacaagtaaagtctttagcccaacttgcgacagcaacctgatcgaaaaatgctcaaataaagaactggtcaatacatacgacaatacgctagtttatactgatcgtatgctgagcaaaactattgaactgttgcaacgttattccgggatgcgtgacgttgctatgatatatctttctgatcatggagaatcgctgggggaaagcggaatatatcttcatggcacaccatatattattgcccccaatgaacaaacacacatcccgatgtttatgtggttttcgtcttcattcgcgcagcattccaaattaaatctagaatgcctgaccggtaatgccgacaaacaatacagtcatgataatttttatcattcaatacttggtctcttcaacgtaaaaaccagtgtatataaaccggagttagatatgtttactctatgtcgacaatctgaccacacaccactgtcttccgcagttgtaagagagaaaacagatgggaatggttag |
